# Local perspectives of the ability of HIA stakeholder engagement to capture and reflect factors that impact Alaska Native health

**DOI:** 10.3402/ijch.v73.24411

**Published:** 2014-06-03

**Authors:** Jen Jones, Nancy A. Nix, Elizabeth Hodges Snyder

**Affiliations:** Department of Health Sciences, University of Alaska Anchorage, Anchorage, AK, USA

**Keywords:** Indigenous, health impact assessment, community engagement, Alaska, wellbeing

## Abstract

**Background:**

Health impact assessment (HIA) is a process used to inform planning and decision making in a range of sectors by identifying potential positive and negative health effects of proposed projects, programs, or policies. Stakeholder engagement is an integral component of HIA and requires careful consideration of participant diversity and appropriate methodologies. Ensuring that the engagement process is able to capture and address Indigenous worldviews and definitions of health is important where Indigenous populations are impacted, particularly in northern regions experiencing increases in natural resource development activities on Indigenous lands.

**Objective:**

Investigate local participant perspectives of an HIA of a proposed Alaska coal mine, with a focus on the ability of the HIA process to capture, reflect, and address health concerns communicated by Alaska Native participants.

**Design:**

A qualitative approach guided by semi-structured interviews with purposeful sampling to select key informants who participated in the coal mine HIA stakeholder engagement process.

**Results:**

Qualitative data identified three key themes as important from the perspective of Alaska Native participants in the Alaska coal mine HIA stakeholder engagement process: (i) the inability of the engagement process to recognize an Indigenous way of sharing or gathering information; (ii) the lack of recognizing traditional knowledge and its use for identifying health impacts and status; and (iii) the inability of the engagement process to register the relationship Indigenous people have with the environment in which they live. Issues of trust in the HIA process and of the HIA findings were expressed within each theme.

**Conclusions:**

Recommendations derived from the research identify the need to acknowledge and incorporate the history of colonialism and assimilation policies in an HIA when assessing health impacts of resource development on or near Indigenous lands. These historical contexts must be included in baseline conditions to understand particular vulnerabilities and potential health risks and impacts. Further, HIA practitioners should recognize the range of definitions for “health” and demonstrate this recognition throughout the stakeholder engagement process, as well as in the HIA recommendations and suggested mitigations.

A health impact assessment (HIA) uses a series of tools and procedures to judge the potential effects of a policy, program, or project on human health as well as identify existing health issues and the distribution of impacts on different sectors of the population (1–3). An HIA is used to inform planning and decision making in a range of sectors including resource development, and “can facilitate adjustment to a proposed policy [or project] in order to mitigate the negative and maximize the positive impacts” (4), p. 1123). The application of HIA has increased around the world over the past two decades (5), and while recommended in the United States, it is not required. Alaska has set a precedent by developing a state HIA Program as a best practice to responsible development (6).

Stakeholder engagement is an integral component of an HIA, offering an opportunity to disclose potential impacts of a project and/or receive feedback from citizens on health issues (7–9). Stakeholders are people or entities that may be affected by a proposed project and include community-based organizations, residents, small businesses, and academia, as well as industry and big businesses (8,10). Proponents of stakeholder engagement (2,3,7) suggest individuals and communities impacted by a project or program should have the opportunity to inform or contribute a voice to the design and implementation of the project or program (8,11), and HIA best practice manuals (9,10,12,13) encourage stakeholder participation as a method to “improve the quality and relevance of the [assessment] findings” (12, p. 32). Stakeholder engagement is an opportunity to gauge public concern, prevent or address existing or potential community conflicts related to a project that may arise during the permitting process (as is often the case with resource development) and support relationship building between proponents and affected communities (2,8,14). Attention can also be drawn to community priorities and values (13). As HIAs most often rely on existing health status information and public health expertise (2,3), stakeholder comments can offer additional information often not readily accessible through traditional forms of evidence-based research (8). During focus groups and/or interviews, stakeholders can share anecdotal information and local history, as well as traditional and cultural knowledge that inform local health issues related to the project under assessment (8,9,13). This is particularly, but not exclusively, germane for northern communities in which Indigenous health concerns or issues related to resource development are being highlighted as industry vies for access to sites on traditional land or near Indigenous communities (15,16). This tasks HIA practitioners with understanding the “complex relationship between social, spiritual, economic, political and cultural determinants with the natural environment” (15, p. 59), key to understanding Indigenous health.

Yet for all the documented and/or theoretical benefits of engaging stakeholders, such as improved health outcomes, environmental sustainability, economic growth, and/or mitigation of existing hazards (2,12,17), how the engagement process is practiced varies across jurisdictions (18). Often gaps exist between the professional rhetoric of engagement and the reality of practice (7). Practitioners and stakeholders may perceive the engagement process differently, resulting in stakeholders questioning their contributions and the subsequent impact on the assessment process. Differences in opinion of the success of stakeholder engagement may be an outcome of the engagement process being challenged with accurately translating comments and information gathered during the consultation process and/or responding to them in a meaningful and timely manner. Local participation may also be challenged when residents perceive unfair and inequitable use of comments or information or the lack of information reflecting cultural and historical context in the HIA (14,19).

Stakeholders themselves are diverse, which can affect the tone or success of the engagement process and influence a participant's experiences. Different belief systems, values, education, and employment among the stakeholders create challenges for practitioners to develop and implement an engagement process that responds to stakeholder diversity (20). Stakeholders may or may not understand how information from an HIA informs a decision or policy or see the connection between health impacts, mitigations and recommendations. Individuals or groups who actually participate in a stakeholder engagement process may also have varying levels of interest in the process, different reasons for participating, and intangible or unrealistic, in practice, ideas to mitigate impacts (21). This compounded with a range of HIA practitioner experiences, time available, access to funding, type of HIA used and/or type of project under assessment results in a diversity of stakeholder engagement experiences within and between HIAs (13,22).

A study of a previously conducted HIA on proposed mining activities for the Wishbone Hill Mine (WHM) in South Central Alaska offers an opportunity to examine Alaska Native stakeholder perspectives of the stakeholder engagement process used for a resource development HIA. An improved understanding of participant perspectives on the ability of HIAs and the stakeholder engagement process to capture, reflect, and address factors that impact the health of citizens can inform continued improvement of stakeholder engagement activities and foster stakeholder trust in the findings of an HIA. This paper focuses on participant perspectives of the WHM HIA stakeholder engagement process, particularly Alaska Native perspectives of the ability of the HIA stakeholder engagement process to recognize worldviews and knowledge systems that inform an Indigenous definition of health and wellness (23). The research findings are from a larger research project that included both Native and non-Native participants and offered recommendations to support better engagement and involvement of participants, as well as improved utilization and communication of findings in the HIA stakeholder engagement process.

## Wishbone Hill Mine HIA case study

**Map. 1:**
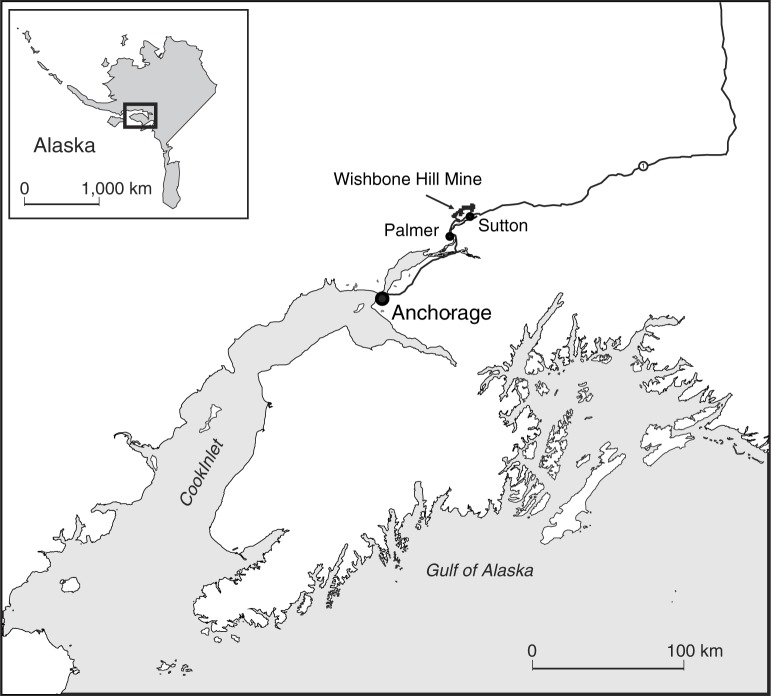
Locator map.

Alaska has the only state HIA program in the United States, and is a leader in conducting resource development HIAs (24). In 2011, the Alaska HIA Program conducted an HIA on the Wishbone Hill Mine, a coalmine seeking development approval and located in the Matanuska-Susitna (Mat-Su) Valley north of Anchorage, Alaska (25) (see Map 1). Of the people living in the Mat-Su Valley, 37,229 were identified as being potentially impacted by the mine development (25). The “impact zones” included eight communities in addition to individuals living adjacent to the mine site, along the transportation route, and within 5 km of the port to be used for exporting coal extracted from the proposed mine (25). Groups invited by the Alaska HIA Program to participate in the HIA stakeholder engagement process were selected from communities within zones 1 and 2 which include the town of Sutton, the community located nearest to the proposed mine site (25).

About 7% of the population living within the two impacted zones self-identified as Alaska Native (25). The proposed mine is located in the traditional territory of the Athna Athabascan people and the governing Chickaloon Village Traditional Council (CVTC) (26). Similar to other Indigenous groups, the Alaska Native peoples of the area continue to address the impacts of colonialization and assimilation policies historically forced upon their peoples (26).

In August and September 2011, the Alaska HIA Program asked community leaders representing citizens in zones 1 and 2 to gather groups of 10 or fewer individuals to participate in a series of stakeholder meetings (25). Seven meetings were held with five different groups. The CVTC, one of the five groups consulted, was consulted twice – the first open to all CVTC members and the other specifically for Elders. Additionally, a subgroup of community leaders of one of the stakeholder groups participated in a preparatory meeting. The general public of the region was invited to submit written comments directly to the Alaska HIA Program (25).

## Research design and methods

Using qualitative description (QD), a method of naturalistic inquiry (27), this research sought to seek straightforward descriptions of the experiences (26,27) of individuals who participated in the WHM HIA stakeholder engagement process. QD is useful when working with community groups as it aims to describe the experiences of participants without theorizing findings (28) or adding additional meaning (29). QD was used to ensure that subsequent recommendations reflected suggestions and feedback from the research participants.

The research took place over 9 months with participant interviews occurring 4 months after the release of the draft WHM HIA (25). Preliminary findings were shared during a public community presentation, at which time additional comments were solicited. The CVTC Chief and Council were also provided an opportunity to share additional comments during a separate PowerPoint presentation. Ethics approval for the research was obtained through the University of Alaska Anchorage Institutional Review Board. In recognition of conducting research involving Indigenous groups, permission from the CVTC to interview citizens who had participated in the WHM HIA stakeholder meetings was sought and granted, with the request from CVTC to review the findings prior to publication.

### Recruitment and sampling

The research used purposeful sampling to ensure representation from each of the five stakeholder groups listed in the draft WHM HIA (25) (see Table I). Research participants were recruited from lists provided by community leaders representing each of the stakeholder groups. While the draft WHM HIA did not identify the total number of participants nor the number of participants representing each of the stakeholder groups invited to participate in the HIA consultation process (25), participants in this research suggested that focus groups consisted of three to 15 participants. A single eligibility criterion was established for participation in this research: attendance at one of the seven WHM HIA stakeholder meetings. Research participants included both Native and non-Native HIA participants. Only participants attending the CVTC stakeholder meetings identified as representing local Alaska Native concerns and this paper focuses primarily on contributions from those participants.

**Table I:** Interview participant representation from WHM HIA focus groups and research

Designated focus groups from the WHM HIA (2012)
Buffalo/Soapstone Focus Group
Chickaloon Focus Group
Members of Castle Mountain Coalition Focus Group
Chickaloon Village Traditional Council Focus Group
Sutton Focus Group
n=12

### Data collection and analysis

Twelve open-ended semi-structured interviews were conducted that examined stakeholder perspectives of the WHM HIA stakeholder engagement process and its ability to capture, reflect and address health concerns of citizens situated in the impact zones. The 10 in-person and two by telephone interviews ranged from 35 to 80 minutes in length and were digitally recorded and transcribed verbatim. All identifiers were removed and a random letter was assigned to each research participant. The interview questions explored participants’ concepts of health, their experiences with the WHM HIA engagement process and their opinions on the ability of the WHM HIA to consider or address their understanding of health and wellbeing. The research also queried research participants about factors that prevented or enhanced full participation in the HIA stakeholder engagement process.

Transcribed interviews were coded by themes that emerged from the data (30). Using Microsoft Word tables to organize the data (31), attention was also given to the context of the coded segments (an important component of QD) by attaching larger narrative units that included immediately preceding/following participant remarks (30).

## Results

Five major themes emerged from the interviews and 18 subcodes were developed to further refine the data and highlight key findings (see Table II). As this paper focuses on Alaska Native perspectives of HIA stakeholder engagement, the following results centre on the ability of the HIA to capture and reflect Indigenous issues shared during the engagement process.

**Table II:** Primary codes and subcodes for key informant interview data

Primary code	Subcode
Experience of process	Expectation of HIA
	Confidence in process
	Knowledge of HIA
	Reasons for participating
	Objectivity of the process
	Trust
Use and source of information	Weight of information
	Cultural knowledge
	Type of HIA
	Data gaps
	Community context
Participant conceptualization of health	Defining health
	Translation of health
	Consideration of Alaska Native health
Participation	Access to stakeholder meetings
	Fear of retribution
	Cultural issues & racism
Prospects for participation in an HIA	Supports & recommendations

### Experiences of the process

Issues of trust resonated throughout all of the interviews conducted for this research, not just with Alaska Native participants. Yet for Alaska Native participants, trust was uniquely contextualized as needing to be a reciprocal arrangement between HIA practitioners and stakeholders in order to safely address historical mistrust resulting from past assimilation and colonial policies experienced by the Indigenous populations in the area (Wade L, written communication, January 2013). Alaska Native participants noted that the WHM HIA engagement process was not always emotionally safe and a lack of cultural awareness demonstrated in the HIA stakeholder engagement meetings contributed to the perspective that Elders and family members were being re-victimized.

We had an Elder, who was talking about boarding schools and being at boarding school and what that was like. For the rest of the family to hear that is kind of traumatic, because they didn't have to go through that. (Participant Y)

Alaska Native participants also spoke of the need for HIA practitioners to recognize the importance of listening and learning to decipher stories told by Elders as well as understand the significance of cultural practices such as sharing food and gifts when traditional information is disclosed during an engagement process. The role of the HIA practitioner and the methods used in the WHM HIA stakeholder engagement process differed from what Alaska Native participants expected or felt necessary to draw out anecdotal or historical health information relevant to the permitting and operation of the WHM.

The second time [the HIA practitioner] came to an Elder's lunch and brought some other [person] with them. That's when I was really quite offended mainly by the other person brought to the Elders lunch and it's my recollection that I sat across from them. I don't even remember what [they] said, all I know is [they] were not very nice. [The HIA practitioner] appeared to be rude and mean and disrespectful and I didn't even stay. I think I may have eaten my lunch but I did not want to be around [them]. I didn't want to hear what [they] had to say, I just wanted to be gone. (Participant C)

### Use of knowledge and information

Some Alaska Native participants stated that the information collected during the stakeholder meetings and published in the draft WHM HIA (25) did not accurately represent the status and determinants of Alaska Native health. It was also noted that the HIA was not able to characterize or incorporate the historical context of local Native peoples’ experiences as they related to the proposed mine. Participants expressed concern that shared stories about the change to traditional lifestyles, forced relocation, and mandatory boarding schools did not appear in the draft HIA. In communications with the Alaska HIA Program, CVTC staff members identified the availability of local Elder ethnographies on traditional and current use of the area to address the difficulty of locating documentation related to traditional knowledge and local history (Wade L, written communication January 2013).

Participants also believed comments from the different stakeholders groups were weighted or used differently in the assessment and that this would contribute to the outcome of the assessment process.

Throughout the [HIA] one of the project managers [for the coalmine] was quoted being an expert in all these different things and yet there's really nowhere in the [HIA] anything quoted by our Elders or any of the community members that contributed to the traditional knowledge. So really there was already a discounting felt in just how information was treated. (Participant Y)

### Concept of health

When asked about concept of health, Alaska Native participants spoke of the relationship between the environments in which they live, their culture and the role these factors play in protecting and promoting the health of their people. In order to be healthy, as one participant stated, “the environment has to be healthy” (Participant J). Another Alaska Native participant spoke of the impacts of colonial history on health.

I'm learning about my native traditions and health and about how to take care of myself through the native traditions as an adult. I didn't learn these things as a child. My mother and my father were both in the boarding school system. The things they did teach me were things that weren't visual. They weren't things that you acknowledged … they showed me by doing …. There are lots of different ways I guess to be healthy in my perspective, one is to live in traditional manner with the Ahtna beliefs. (Participant F)

Nearly all of the research participants defined health as a holistic state “that included mind, body and spirit” (Participant J). When asked if their concepts of health were reflected in the HIA, Alaska Native participants expressed concern that little attention was given to addressing the complex relationship Alaska Native peoples have with their environment and the implications an operating mine would have on this connection. Participants felt this was a result of the lack of recognition of an Indigenous worldview and past experiences.

If they would include the history and what happened, [the HIA practitioners] may gain a better understanding. That might be a start if they would care. Just to care about the care of the land. The care of the people. If they would try to show they cared about what happened in the past and this is why we don't want it to happen again. (Participant C)

## Discussion

While Alaska Native participants in this research recognized the potential benefits of conducting an HIA of a proposed resource development project, insufficient practitioner attention to cultural and traditional knowledge, experiences, and expectations during the stakeholder engagement process appears to have impacted Alaska Native participant perspectives of the ability of an HIA to address Indigenous health issues and concerns. Alaska Native participants expressed concern about the ability of the HIA stakeholder engagement process to translate the unique and complex relationship between the environment, the protective factor of culture, and the legacy of colonialism and assimilation policies (32). This research suggests that for effective HIA stakeholder engagement of Indigenous populations to take place, the difficult conversation addressing the impacts of assimilation policies, systemic racism and the impacts on community health and individual wellbeing is necessary and should be sensitively conducted. Determinants of Indigenous health cannot be addressed without understanding the historic mistrust between Indigenous populations and governments (32,33) nor the legacies of previous mines or development in the area and/or their subsequent pressure to traditional lifestyle, as experienced in recent history such as forced relocation and mandatory boarding school (26).

While stakeholder engagement provides an opportunity to gather local information, it is necessary to consider how this information is acquired. Gaining access to a community's local information, history or politics requires navigating and negotiating entry into the community, building trust and working with local representatives to ensure the process of soliciting information is respectful of local customs and sensitivities. Securing trained support staff during meetings where sensitive information is shared or heard for the first time by other community members may demonstrate respect and understanding of existing intergenerational trauma as well as address concerns raised about cultural safety and sensitivity. In addition, hosting more than one meeting with Indigenous groups, as was done for the WHM HIA, and applying community driven facilitation approaches could support an engagement process that facilitates an environment in which participants feel safe to share personal histories or relevant traditional knowledge (16,33).

Factors that influence Indigenous health are complex and their linkages are not always orderly or linear, and the reality is Indigenous knowledge and history is often difficult to access, as it is not always found in a written format (16). This lack of access to physical documentation that can be used to verify, confirm, or complement stakeholder input can be a challenge for HIA practitioners. In this study, participants expressed concern that information collected from non-traditional sources (e.g. Elder stories) were ignored or considered marginal and resulted in information relevant to local Alaska Native health being overlooked. The use of Indigenous approaches to knowledge transfer (16) as well as new or innovative approaches to sourcing data or including information or anecdotal stories that provide insight into the connection between culture, relationship with the land and the impacts of colonialism (16,23,32) on health is necessary for HIA to resonate, have relevance and be constructive for policy and decision makers in the north.

For HIA practitioners to better understand the local environment and health status of its inhabitants, and for stakeholders to trust HIA as an effective assessment tool in the context of northern resource development, further work is required to identify and/or develop methods that can support analysis of the intersection of a proposed project, the complexities that inform the health of northern Indigenous populations and the variety of stakeholders who will participate in an engagement process.

## Conclusion

This research aimed to characterize Alaska Native perspectives of an HIA engagement process and drew from results of a larger study that included both Native and non-Native participants. The research was not an evaluation of the WHM HIA, but rather sought out participant experiences to inform recommendations for more meaningful engagement with Indigenous populations. Illuminating and characterizing these perspectives offers an opportunity to reflect on how the HIA stakeholder engagement process may, or may not, be capturing the necessary information to effectively examine and assess the full range of potential positive and negative impacts of a project on Indigenous health. Five recommendations are offered as a starting point to better engage Indigenous peoples in the HIA process.

- Adopt community driven facilitation approaches to prevent and/or mitigate additional trauma and re-victimization of Indigenous participants during the engagement process.
- Provide training, time and funding to support this approach, which are all necessary to address the issues of intergenerational trauma in a respectful manner and to build and ensure trust during the stakeholder engagement process.
- Recognize the impact of assimilation policies and colonialism on current health status and demonstrate this recognition in the recommendations and mitigations.
- Recognize the range of definitions for “health” and demonstrate this recognition in the recommendations and mitigations.
- Engage Indigenous organizations/communities to further develop methodologies that are capable of accommodating a broad definition of health and range of impacts from an Indigenous perspective.

The recommendations are consistent with those of Indigenous scholars (19) who suggest that engagement with Indigenous populations be built upon trust through acknowledging historical experiences with research and health issues; recognizing Indigenous sovereignty; understanding Indigenous diversity and its implications; planning for extended timelines; interpreting data within the cultural context; and utilizing Indigenous ways of knowledge (16,19).

As HIA becomes increasingly recognized as a tool to characterize potential health impacts of natural resource development projects in the north, capturing the confidence of northern Indigenous populations by demonstrating that the tool is able to objectively examine the impacts while recognizing the distinct cultural differences that exist in northern populations is important. While this is the challenge for any engagement process, for HIAs to delay prioritizing this task puts at possible risk the confidence northern Indigenous populations have in the ability of an HIA to effectively address the complex health and cultural impacts and develop recommendations to mitigate negative impacts associated with a proposed project.

## References

[1] European Centre for Health Policy (1999). Health impact assessment: main concepts and suggested approach. http://www.apho.org.uk/resource/view.aspx?RID=44163.

[2] Health Canada (2004). Canadian handbook on health impact assessment: volume 1: the basics. http://www.publications.gc.ca/collections/Collection/H46-2-99-235E-1.pdf.

[3] Wernham A (2011). Health impact assessments are needed in decision making about environmental and land-use policy. Health Aff.

[4] Parry JM, Kemm JR (2005). On behalf of all participants of the Evaluation of Health Impact Assessment Workshop. Criteria for use in the evaluation of health impact assessments. Public Health.

[5] Winkler MS, Krieger GR, Divall MJ, Cisse G, Wielga M, Singer BH (2013). Untapped potential of health impact assessment. Bull World Health Organ.

[6] State of Alaska Department of Health and Social Services Alaska health impact assessment (HIA) program. http://www.epi.alaska.gov/hia/.

[7] Kearney M (2004). Walking the walk? Community participation in HIA: a qualitative interview study. Environ Impact Assess Rev.

[8] Stakeholder Participation Working Group in the 2010 HIA in the Americas Workshop (2011). Best practices for stakeholder participation in health impact assessment. http://www.healthimpactproject.org/resources/document/Guide-for-Stakeholder-Participation.pdf.

[9] International Finance Corporation (2009). Introduction to health impact assessment. http://www.ifc.org/wps/wcm/connect/topics_ext_content/ifc_external_corporate_site/ifc+sustainability/publications/publications_handbook_healthimpactassessment__wci__1319578475704.

[10] State of Alaska HIA Program, Department of Health and Social Services (2011). Technical guidance for health impact assessment (HIA) in Alaska. http://www.epi.hss.state.ak.us/hia/AlaskaHIAToolkit.pdf.

[11] Lockie S, Franetovich M, Sharma S, Rolfe J (2008). Democratisation versus engagement? Social and economic impact assessment and community participation in the coal mining industry of the Bowen Basin, Australia. Impact Assess Proj Apprais.

[12] International Council on Mining and Metals (2010). Good practice guidance on health impact assessment. http://www.icmm.com/library/hia.

[13] Bhatia R (2010). A guide for health impact assessment. http://www.cdph.ca.gov/pubsforms/Guidelines/Documents/HIA%20Guide%20FINAL%2010-19-10.pdf.

[14] Ali S, O'Callaghan V, Middleton JD, Little R (2009). The challenges of evaluating a health impact assessment. Crit Public Health.

[15] Kwiatkowski RE, Tikhonov C, Peace DM, Bourassa C (2009). Canadian Indigenous engagement and capacity building in health impact assessment. Impact Assess Proj Apprais.

[16] Kwiatkowski RE (2011). Indigenous community based participatory research and health impact assessment: a Canadian example. Environ Impact Assess Rev.

[17] Tamburrini A, Gilhuly K, Harris-Roxas B (2011). Enhancing benefits in health impact assessment through stakeholder consultation. Impact Assess Proj Apprais.

[18] Tindana PO, Rozmovits L, Boulanger RF, Bandewar SVS, Aborigo RA, Hodgson AVO (2011). Aligning community engagement with traditional authority structures in global health research: a case study from northern Ghana. Am J Public Health.

[19] Christopher S, Watts V, McCormick AK, Young S (2008). Building and maintaining trust in a community-based participatory research partnership. Am J Public Health.

[20] Negev M (2012). Knowledge, data and interests: challenges in participation of diverse stakeholders in HIA. Environ Impact Assess Rev.

[21] Elliott E, Williams G (2004). Developing a civic intelligence: local involvement in HIA. Environ Impact Assess Rev.

[22] Strauss H (2011). Involving the Finnish public in nuclear facility licensing: participatory democracy and industrial bias. J Integr Environ Sci.

[23] Friendship KA, Furgal CM (2012). The role of Indigenous knowledge in environmental health risk management in Yukon, Canada. Int J Circumpolar Health.

[24] Wernham A (2009). Building a statewide health impact assessment program: a case study from Alaska. Northwest Public Health.

[25] NewFields Companies (2012). Health impacts assessment for proposed coal mine at Wishbone Hill, Matanuska-Susitna Borough Alaska. http://www.epi.hss.state.ak.us/hia/WishboneHillDraftHIA.pdf.

[26] Athabascan Nation Chickaloon Village Brief history. http://www.chickaloon.org/about/brief-history.

[27] Sullivan-Bolyai S, Bova C, Harper D (2005). Developing and refining interventions in persons with health disparities: the use of qualitative description. Nurs Outlook.

[28] Neergaard MA, Olesen F, Andersen RS, Sondergaard J (2009). Qualitative description—the poor cousin of health research?. BMC Med Res Methodol.

[29] Sandelowski M (2000). Whatever happened to qualitative description?. Res Nurs Health.

[30] Milne J, Oberle K (2005). Enhancing rigor in qualitative description: a case study. J Wound Ostomy Continence Nurs.

[31] La Pelle N (2004). Simplifying qualitative data analysis using general purpose software tools. Field Methods.

[32] Mindell J, Biddulph J, Taylor L, Lock K, Boaz A, Joffe M (2010). Improving the use of evidence in health impact assessment. World Health Organization. Bulletin of the World Health Organization.

[33] Richmond CAM, Ross NA (2009). The determinants of First Nation and Inuit health: a critical population health approach. Health Place.

